# Role of Environmental Degradation, Institutional Quality, and Government Health Expenditures for Human Health: Evidence From Emerging Seven Countries

**DOI:** 10.3389/fpubh.2022.870767

**Published:** 2022-03-24

**Authors:** Jiping Wei, Syed Rahim, Shizhen Wang

**Affiliations:** ^1^School of Management, Chengdu University of Traditional Chinese Medicine, Chengdu, China; ^2^Pakistan Institute of Development Economics, Islamabad, Pakistan; ^3^School of Economics and Management, Anqing Normal University, Anqing, China

**Keywords:** human health, environmental degradation, regulatory quality, health expenditure, human capital, quantile regression

## Abstract

The current study investigates the association of various economic, non-economic, governance, and environmental indicators on human health for seven emerging economies. Covering the period from 2000Q1 to 2018Q1, this study uses various panel data approaches for empirical estimations. The data is found first-order stationary. Besides, the panel slope is heterogeneous and cross-sectional dependence is present. Further, the cointegration association is found valid among the variables. Therefore, panel quantile regression is used to determine the long-run impact of each explanatory variable on human health at four quantiles (Q_25_, Q_50_, Q_75_, and Q_90_). The estimated results asserted that economic growth, government health expenditure, and human capital significantly reduce human health disasters like malaria incidences and cases. At the same time, greenhouse gas emissions and regulatory quality are significantly and positively correlated to human health issues in emerging economies. Moreover, mixed (unidirectional and bidirectional) causal associations exist between the variables. This study also provides relevant policy implications based on the empirical results, providing a path for regulating various economic, environmental, and governance sectors. Effective policy implementation and preventive measures can reduce the spread of diseases and mortality rates due to Malaria.

## Highlights

- The association of various economic, non, economic, governance indicators, and human health are examined.
- Seven emerging economies are considered as a panel from 2000 to 2018.
- Panel data approaches and quantile regression is used to obtain the empirical results.
- Greenhouse gas emissions and regulatory quality adversely affect human health.
- Health expenditures, economic growth, and human capital significantly positively influence human health.
- Mixed causal associations are found existing between the study variables.

## Introduction

Rapid expansions and economic development have deteriorated the environmental quality (1, 2). Greenhouse gases and carbon emissions are the main culprits of environmental deterioration worldwide. Researchers and scholars argued that global warming is directly or indirectly associated with the recurrence of the Malaria epidemic, which is again a growing concern internationally (3, 4). About 80% of the world's global warming is due to consumed energy, ~75% is due to greenhouse gas emissions causing abrupt climate changes (5). Severe climate changes have caused various diseases and health disparities The global temperature varies with acute effects on human health (6, 7). The impact of global warming depends on the human population (host) and infectious agents. It is a gradual process that has serious and harmful consequences with time. The environmental changes trigger and lead to a shift in disease patterns (8, 9). Over the past years, mortality and diseases have risen due to environmental pollution but also it is a hurdle in sustainable development (10). The Malaria outbreaks have befallen many temperate areas of the world. According to the research of the World Health Organization (WHO) and others, Malaria is a widely spreading infectious disease from Greenhouse gases. In 2020, half of the world's inhabitants were at risk of the infectious disease; Malaria. Approximately 241 million cases of Malaria were informed in 2020 and 227 million cases in the year 2019, while estimated deaths were 6,27,000 worldwide in 2020 (11). Additionally, the WHO reports that it is expected that the climate conditions may threaten some regions of the world, causing the increasing transmission of Malaria by the year 2050.

Lately, several researchers have studied the influence of environmental degradation on the public's health. The interaction of human health with the environment is a widely researched topic, and many scholars have proven the significant risks toward human health (12). In an exploratory analysis in the case study of Canada, the authors observed a significant relationship of the environment with public health expenses. The association of human health with the environment is an extensive research issue. Many authors observed a significant association of the environment with public health and its financing (13). Escalating environmental degradation has raised the negative influence on health and positive association with institutional quality and health expenses (14). Institutional quality is important because it has significant benefits for environmental enhancement in many developed economies by reducing Carbon emissions (15, 16). Increasing healthcare problems create a burden on governmental expenditure. The major cause of environmental degradation is greenhouse (GHG) and carbon dioxide emissions (17–19). These emissions are not harmful to human health but also affect economic activity adversely (13, 20). The findings of the notable studies indicated that environmental degradation negatively influences people's health. Infectious and other respiratory diseases are spread through carbon and Greenhouse gas (GHG) emissions worldwide. Due to this, the world has adverse impacts on human lives by reducing life expectancy. Health is a national asset that plays a significant role in the economy's prosperity. It governs the human capital, an imperative factor for the economy's growth. The role of government and institutions is essential in delivering the finest healthcare facilities and protecting the environmental quality. An adequate health budget is essential for public health improvement. Government, public institutions, renewable energy consumption, and efficient regulatory environmental laws can help sustainability. Miao et al. (21) revealed that renewable energy and globalization (financially) contribute to the quality of the environment.

The article aims to explore the role of GHG, Government Health expenditures, and Institutional quality for Malaria control or Malaria spread. Numerous authors and researchers in the existing literature have discussed the influence of GHG on public health, while some indicated that GHG helps in the spreading of infectious diseases. However, neither of the studies has explored the role of GHG in spreading or controlling infectious diseases. First, the present study examines the role of greenhouse gases emissions, heath expenses of the government, and institutional quality for control of Malaria in emerging seven economies. These are three BRICS countries, i.e., Brazil, India, and China, including the other emerging countries, i.e., Mexico, Indonesia, Turkey, and Bangladesh, correspondingly. The study aims to bridge the gap by analyzing the Malaria spread or control in these emerging economies. Second, the study adds multifold directions by scrutinizing the Malaria spread on two models. In the first Model, the impact of explanatory variables is analyzed over the incidence of malaria cases per 1,000 population size. In the second Model, the number of Malaria cases reported is used as the dependent variable for the study. This is a significant contribution in the study investigating the role of greenhouse gases on health proxying Malaria control or incidences; a pioneer study in the prevailing academic literature.

The rest of the article is organized as follows. The next section briefs about the literature review for a deep understanding. Section Data and Methodology is about the data and methodology that is used for research. Then, results with discussions and conclusions are elaborated in Sections Results and Discussions and Conclusions and Policy Implication of the article, respectively.

## Literature Review

This segment deals with the literature background and some empirical evidence related to the research in terms of variables and their inter-relationship to the study. The first sub-section is about the background though the second sub-section is about the literature about environmental degradation, institutional quality, and health expenditures for Malaria spread across countries. Then at the end of this segment, the research gap is briefed.

### Literature Backdrop

The existing literature is focused on two specific fields of research. First, the interrelationship of environment changes and health of the public in which the climate has increased cases of health issues, thereby increasing the expenditures on public health and health reforms. Poor health influences the economic performance of the country and cumulative national income (22, 23). The second research area is about carbon emissions and green climate. The research and development in carbon emissions and green technology have gained pace since it requires a massive budget, research, and technological advancement. The world is focusing on carbon-efficient technologies for a greener and cleaner environment for eliminating pollution that affects public health (24, 25). The findings of the novel studies indicated that environmental degradation negatively influences the health of the people in 17 MENA (Middle Eastern and North African) countries. It can be enhanced by good institutional quality with effective environmental laws (26). The poor environment is linked with health disparities and diseases. Many diseases like cancer, Malaria, premature death, and other respiratory diseases are spread through carbon and Greenhouse gas (GHG) emissions. Tyagi et al. (27) observed an overview of studies in the literature on environmental degradation causes. The major consequences of poor quality of the environment are the depletion of natural resources (quality and quantity). They also investigated that this, in turn, affects human health worldwide because of the interconnection with each other. Patz et al. (28) inspected that changing climatic conditions have a significant influence on the public's health. There are physical, chemical, and biological impacts of climate variability such as extreme weather conditions, drought, floods, acid rains, exposure to ozone, infectious diseases cycles (annually), and other vector-borne diseases (Malaria and Dengue) and waterborne diseases (Cryptosporidiosis and Diarrhea). Additionally, it also impacts the productivity of food, especially in those agro-based developing countries based on livestock and farming. In agreement with the notable research for the Chinese economy, the authors studied the dynamic association between economic change, public health, and environmental effluence. They scrutinized the association in 30 provinces of China using panel data. They confirmed the harmful impact of environmental pollution on the public's health that not only affects the GDP per capita but also becomes a hurdle in promoting the growth of the economy (29). Moosa and Pham (30) inspected a positive and significant impact on health expenditures by environmental degradation. They applied the Autoregressive distributive lag and cointegration model to explain the bivariate association, elaborated with the Environmental Kuznets (EKC) hypothesis. They further observed that the relationship could be positive or negative liable on the countries per capita income. Real income harms the environmental quality in both the long and short run (31). Moreover, environmental quality significantly affects health expenditures recognized in the case of MENA states. Such as, carbon and GHG emissions are proportionally related to health expenses because increasing emissions cause expenditures to rise due to health deterioration of the people (32). Furthermore, Edeme et al. (33) applied the Johansen Cointegration and Vector error correction model (VECM) and noticed that environmental factors like carbon emissions influence the public's health. They also suggested that government needs to implement effective programs for carbon emissions to mitigate the effects on the health of individuals of Nigeria.

### GHG Emissions, Health Expenditure, and Institutional Quality in Malaria Spread

The increasing accumulation of greenhouse gas in the earth's atmosphere has changed the environment of the atmosphere. Numerous health analysts observed that the changing temperature conditions have adverse health outcomes. Infectious diseases are spread directly from the source, such as water-foodborne diseases and vector-borne diseases; triggered by climate change. Malaria is one of those diseases caused by sensitivity toward climatic factors. It is a temperature-sensitive illness that occurs due to globalization and the absence of proper public health infrastructure (34). Rogers and Randolph (35) predicted a warmer world with the spread of Malaria in the future across countries worldwide. However, the global temperature has been predicted to be between 1.0 and 3.5°C by 2100 (36). Moreover, the variability of the climate is likely to influence the transmission of Malaria due to escalating Greenhouse gas emissions (37). Ermert et al. (38) assessed the spread of Malaria from 1960 to 2000 in Africa. They indicated that climate changes due to GHG emissions significantly spread Malaria. Eckelman and Sherman (39) examined the factors that link GHG emissions with health and increase the disease burdens such as vector-borne diseases like Malaria in the United States. The emissions related to healthcare are inversely linked with the wellbeing of people due to the high pervasiveness of extreme climatic conditions. They suggested that effective institutional laws regarding health and climate can help in mitigating the adverse effects. Ponku (40) examined the effective role of governance is positively significant in enhancing public health, whereas institutional quality aids public health expenses. Method of moments (GMM) for 22 sub-Saharan African economies was applied to examine that the governance and quality of institutions significantly improve the effectiveness of public health expenditures for better health results. Louis and Hess (41) observed that health concerns have been increased in poorer countries regardless of their insignificant contribution to GHG emissions. Ajide and Alimi (42) examined the role of institutions in the case of Africa from the year 1996 to 2016. They discovered that organizational dysfunction has a significantly negative impact on health results. Additionally, the environmental pollutants and institutions negatively affect life expectancy, whereas they have a significant and constructive influence on health expenses. They concluded that effective governance and institutional quality could improve the health of the public and the performance of necessary services (43). Moosa and Pham (30) inspected the proportional impact of environmental degradation on health expenditures. They concluded that an increase in the deterioration of the environment leads to an increase in government health expenses.

### Research Gap

Quite a lot of studies in the literature have discussed the impact of GHG on public health, while few studies indicated that carbon emissions and GHG help spread harmful infectious diseases. While numerous authors and researchers like (12, 36, 39) have discovered and put deep insights into environmental degradation and its harmful impacts on the lives of humans. However, prior studies ignored the role of greenhouse gas emissions in spreading or controlling infectious diseases like Malaria. Hence, the present study bridge this gap by investigating the association of various economic, non-economic, governance, and environmental indicators on human health for seven emerging economies. Additionally, taking into account the role of institutional quality variables and government health expenses to explore the influence of GHG emissions, anew input academically.

## Data and Methodology

### Data and Model Specifications

This study aims to empirically investigate the influence of economic, non-economic, and governance indicators on human health. Therefore, this study adopts seven variables while following the literature as given in Section Literature Review. To comprehensively analyze the influence of such variables on human health, this study uses two variables indicating Incidence of Malaria (MI) and Malaria cases reported (MC). However, the explanatory variables include emissions—captured by greenhouse gas (GHG) emissions, economic growth—indicated via gross domestic growth (GDP), governance or institutional quality is captured by regulatory quality (RQ). Besides, Domestic general government health expenditures (GHE) and human capital (HC) are also examined in this relationship. Data for these variables are obtained from multiple sources, covering the 2000Q1 to 2018Q4 period for seven emergings (E7) economies, including India, China, Indonesia, Bangladesh, Turkey, Brazil, and Mexico. The primary reason for adopting the small sample is the unavailability of data, where the available data on World Bank site is only for 19 years. The variables' specifications and data sources are provided in Table 1.

**Table 1:** Variables specification and data sources.

**Variable**	**Specification**	**Data source**
*MI*	Incidence of Malaria (per 1,000 population at risk)	http://apps.who.int/ghodata/
*MC*	Malaria cases reported	http://apps.who.int/ghodata/
*GHG*	Total greenhouse gas emissions (thousand metric tons of CO_2_ equivalent excluding Land-Use Change and Forestry)	https://www.climatewatchdata.org/ghg-emissions
*GDP*	A monetary worth of all final products and services produced in a certain period (constant 2015 US$)	https://databank.world-bank.org/source/world-development-indicators
*RQ*	Measures views of the government's capacity to establish and enforce solid policies and regulations that foster private sector growth. The estimate is the country's score on the aggregate indicator, ranging from −2.5 to 2.5	https://databank.world-bank.org/source/worldwide-governance-indicators
*GHE*	Domestic general government health expenditure (% of GDP)	http://apps.who.int/nha/-database
*HC*	Refers to the economic worth of a worker's expertise, knowledge, and skills	www.ggdc.net/pwt

Following the study of Farooq et al. (44), this study constructed two models from the above variables, given as:


$$\begin{array}{l} MI_{it}=f\left(GHG_{it},GDP_{it},RQ_{it},GHE_{it},HC_{it}\right)\\ MC_{it}=f\left(GHG_{it},GDP_{it},RQ_{it},GHE_{it},HC_{it}\right) \end{array}$$

These models demonstrate that *GHG*_*it*_, *GDP*_*it*_, *RQ*_*it*_, *GHE*_*it*_, and *HC*_*it*_ are the functions of *MI*_*it*_ and *MC*_*it*_. However, these models could adopt the following econometric form for empirical examination:


(1) $$\begin{array}{r} MI_{it}=\gamma_{0}+\gamma_{1}GHG_{it}+\gamma_{2}GDP_{it}+\gamma_{3}RQ_{it}+\gamma_{4}GHE_{it}\\ \;+\gamma_{5}HC_{it}+\varepsilon_{it} \end{array}$$

(2) $$\begin{array}{r} MC_{it}=\gamma_{0}+\gamma_{1}GHG_{it}+\gamma_{2}GDP_{it}+\gamma_{3}RQ_{it}+\gamma_{4}GHE_{it}\\ \;+\gamma_{5}HC_{it}+\varepsilon_{it} \end{array}$$

Where γ′*s* are the coefficients to be estimated and γ_0_ is the intercept in both the equations. Whereas, *I* and *t* in the subscript show cross-sections and time-period, respectively. Besides, the “ε” is the random error term of the regression model.

### Estimation Strategy

#### Slope Heterogeneity and Cross-Section Dependence

Following the industrial revolution, there was a marked increase in international business and globalization, even though various variables influence an economy and its reliance on other nations. Specialization of one economy in particular commodities or services draws the attention of other economies and nations dependent on these services and products. The primary reason for this dependency is to accomplish numerous aims and objectives specified by governments or states, including social, cultural, financial, technological, economic, and technical purposes. Due to such reasons, one country's economy may exhibit similarities or disparities in some areas compared to other economies. Panel data estimate methodologies such as slope heterogeneity and cross-sectional dependency are used in this work. Whereas, if slope heterogeneity and cross-section dependence are disregarded, the econometric analysis may provide inefficient results (45). As a result, these two-panel data concerns are examined here using the Pesaran and Yamagata (46) slope coefficient homogeneity (SCH) and Pesaran (47) cross-section dependency (CD) tests. The conventional equation for estimating might be as follows with consideration to the SCH test:


(3) $$\begin{array}{r} {\hat{\Delta }}_{SCH}=\sqrt{N{\left(2k\right)}^{-1}}\;\left(N^{-1}Ś-K\right), \end{array}$$

Apart from SCH, this test also evaluates adjusted SCH, which may be expressed in the following standard formula:


(4) $$\begin{array}{r} {\hat{\Delta }}_{ASCH}=\;\sqrt{N}\sqrt{\frac{T+1}{2K\left(T-K-1\right)}}\;\left(N^{-1}Ś-2K\right), \end{array}$$

The test presumes homogenous slopes coefficients as the null proposition, while the alternate hypothesis could be accepted only if the estimates are significant.

Likewise, cross-section dependence cannot be overlooked since it may result in a biased estimate in an economic inquiry (48). The Pesaran (47) CD test is employed in this instance, and the typical formulation is as follows:


(5) $$\begin{array}{r} {\text{CD}}_{Test}=\frac{\sqrt{2.T}}{{\left[N\left(N-1\right)\right]}^{1/2}}\;\overset{N-1}{\underset{i=1}{\sum }}\;\overset{N}{\underset{k=1+i}{\sum }}T_{ik}, \end{array}$$

The under-discussion test is predicated on the independence of panel cross-sections in the chosen panel economies. On the other hand, the alternative hypothesis will be adopted if the estimates are shown to be significant at any of the 1, 5, or 10% levels.

#### Stationarity Testing

According to the empirical estimations of slope heterogeneity and cross-section dependence, the slopes are heterogeneous, and the panel exhibits cross-section reliance. As a result, it is critical to use an estimator that effectively addresses the previously described panel data problem. In this regard, the present investigation used Pesaran's (49) cross-sectionally augmented IPS—termed as the CIPS unit root test. Initially, while considering cross-section dependency, Pesaran (50) advocated for a factor modeling method. The averages of cross-sections are combined in the same manner as the Model's common unobserved components. Pesaran (49) used a similar technique and devised an additional strategy for unit root testing by expanding the Augmented Dickey-Fuller (ADF) regression model to include not only the mean and cross-sectional first difference lags. Even when the panel is imbalanced, i.e., the cross-section and time period are not equal (*N*≠*T*), the said technique addresses the CD problem. The cross-sectional ADF has the following standard equation form:


(6) $$\begin{array}{r} \Delta y_{i,t}=\;\theta_{i}+\beta_{i}^{*}y_{i,t-1}+d_{0}.{\bar{y}}_{t-1}+d_{1}\Delta {\bar{y}}_{t}+\varepsilon_{it}, \end{array}$$

As shown in Equation (6), ${\overset{\bar{}}{y}}_{t}$ is the average of the observations. To account for serial correlation, this expression may be changed by adding the following first differenced lags for *y*_*it*_ and ${\bar{y}}_{t}$:


(7) $$\begin{array}{rc} \Delta y_{i,t}=\;\theta_{i}+\beta_{i}^{*}y_{i,t-1}+d_{0}{\bar{y}}_{t-1}+\overset{n}{\underset{j=0}{\sum }}d_{j+1}\Delta {\bar{y}}_{t-j}+\overset{n}{\underset{k=1}{\sum }}c_{k}\Delta y_{i,t-k}\\ + & \varepsilon_{it}, \end{array}$$

Thus, the CIPS is formed and is used in this research to assess for the existence of a unit root by averaging the *t*-statistics for each unit of the cross-section, referred to as the *CADF*_*i*_, and given as:


(8) $$\begin{array}{r} CIPS=N^{-1}\overset{N}{\underset{i=1}{\sum }}\;CADF_{i}, \end{array}$$

This test (CIPS) proposes the null hypothesis, indicating that the unit root exists in the data, but the alternative hypothesis claims that the data is stationary across time.

#### Panel Cointegration Test

Since each variable has a panel unit root, it is important to investigate the long-run equilibrium between the variables under consideration. In this sense, the current study utilized two-panel cointegration tests, including Kao (51) and Pedroni (52) cointegration tests. The Pedroni (52) test provides estimates for Modified Phillips-Perron *t*, Phillips-Perron *t*, and Augmented Dickey-Fuller *t*. While the Kao (51) cointegration test provides statistical values for Modified Dickey-Fuller *t*, Dickey-Fuller *t*, Augmented Dickey-Fuller *t*, Unadjusted modified Dickey-Fuller *t*, and Unadjusted Dickey-Fuller *t*. The null hypothesis of these tests presumes that no cointegration exists in the variables. However, the statistically significant estimates could lead to the rejection of null and conclude that cointegration exists among the variables.

#### Quantile Regression

Following the slope heterogeneity, cross-section dependency tests, and the cointegration test, we systematically examined the long-run effect of the variables under discussion on both MI and MC using the quantile regression technique developed by Koenker and Bassett (53). The rationale for utilizing quantile regression is because of data's normality, implying that traditional approaches would not generate accurate estimates. Additionally, to prevent the over-and under-estimation bias inherent in these standard methodologies, this research used the quantile regression methodology, which offers the estimated coefficient at each quantile chosen. Since the panel quantile regression accounts for both individual and distributional heterogeneity, it gives detailed information about the connection between the factors under examination (54). Additionally, quantile regression has greater predictive ability than standard regression, which simply offers the average influence of exogenous variables (55). Additionally, the estimator mentioned above is useful due to its management of slope heterogeneity and cross-sectional dependence concerns (56). The previously stated regression equations, namely Equations (1) and (2) may be transformed into panel quantile regression forms using Equations (9) and (10), respectively.


(9) $$\begin{array}{r} Q_{MI_{it}}\left(\theta \;|\;\alpha_{i},\varphi_{t},X_{it}\right)=\alpha_{i}+\varphi_{t}+\varphi_{1,\theta }GHG_{it}+\varphi_{2,\theta }GDP_{it}\\ \;+\varphi_{3,\theta }RQ_{it}+\varphi_{4,\theta }GHE_{it}+\varphi_{5,\theta }HC_{it}\\ \;+\varepsilon_{it} \end{array}$$

(10) $$\begin{array}{rc} Q_{MC_{it}}\left(\theta \;|\;\alpha_{i},\varphi_{t},X_{it}\right)=\alpha_{i}+\varphi_{t}+\varphi_{1,\theta }GHG_{it}+\varphi_{2,\theta }GDP_{it}\\ \;+\varphi_{3,\theta }RQ_{it}+\varphi_{4,\theta }GHE_{it}+\varphi_{5,\theta }HC_{it}\\ + & \varepsilon_{it} \end{array}$$

Whereas, the subscript θ in both equations denote the quantile for each variable while using four quantiles, namely Q_25_, Q_50_, Q_75_, and Q_90_, to experimentally evaluate the effect of GHG, GDP, RQ, GHE, and HC on MI and MC in developing countries.

#### Panel Causality Test

The quantile regression approach provides estimated outputs for each regressor at a certain quantile, but not for their causal link. This study used Dumitrescu and Hurlin's (57) Granger panel causality heterogeneity test to establish causality. This test is more effective and robust in correcting the panel imbalance (*N*≠*T*). Additionally, it handles the heterogeneity of panel data and cross-sectional dependence (58).

## Results and Discussions

This segment of the article deals with research results and discussions. Table 2 shows the Slope Heterogeneity test results; Table 3 represents the cross-section dependence results with their significant values. Table 4 has the unit root tests, whereas Tables 5, 6 shows cointegration outcomes. Tables 7, 8 denote the Quantile regression results for both models, respectively, with the Quantile graphical representation of the Models (1 and 2). Last, of all, Dumitrescu-Hurlin Panel Causality results are displayed in Table 9 of this section.

**Table 2:** Slope heterogeneity.

**Slope heterogeneity test**	**Statistics**
**Model 1**	
$\tilde{\Delta }$	3.692***
${\tilde{\Delta }}^{Adjusted}$	4.646***
**Model 2**	
$\tilde{\Delta }$	3.735***
${\tilde{\Delta }}^{Adjusted}$	4.700***

Significance level is denoted by *** for 1%, ** for 5%, and * for 10%.

**Table 3:** Cross-section dependence.

**Cross-section dependence**	
*MI*	*MC*
8.03***	12.32***
*GHG*	*GDP*
18.77***	19.46***
*RQ*	*GHE*
−0.25	3.30***
**HC**	
17.38***	

Significance level is denoted by *** for 1%, ** for 5%, and * for 10%.

**Table 4:** Unit root testing (49).

**Variables**	**Intercept and trend**	
	**I(0)**	**I(1)**
*MI*	−1.801	−3.828***
*MC*	−1.450	−3.291***
*GHG*	−1.819	−3.756***
*GDP*	−1.223	−2.882**
*RQ*	−1.944	−3.791***
*GHG*	−1.991	−3.990***
*HC*	−2.421	−2.802***

Significance level is denoted by *** for 1%, ** for 5%, and * for 10%. I(0) is for level, and I(1) is for the first difference.

**Table 5:** Cointegration results (Pedroni).

**Statistics**	**Value**
**Model 1**	
Modified Phillips-Perron *t*	2.4302***
Phillips-Perron *t*	−2.0311**
Augmented Dickey-Fuller *t*	−2.5298***
**Model 2**	
Modified Phillips-Perron *t*	2.5562***
Phillips-Perron *t*	−2.4631***
Augmented Dickey-Fuller *t*	−2.1752**

Significance level is denoted by *** for 1%, ** for 5%, and * for 10%.

**Table 6:** Cointegration results (Kao).

**Statistics**	**Value**
**Model 1**	
Modified Dickey-Fuller *t*	−4.7081***
Dickey-Fuller *t*	−2.3708***
Augmented Dickey-Fuller *t*	−2.9419***
Unadjusted modified Dickey-Fuller *t*	−4.7207***
Unadjusted Dickey-Fuller *t*	−2.3741***
**Model 2**	
Modified Dickey-Fuller *t*	1.5645*
Dickey-Fuller *t*	1.5749*
Augmented Dickey-Fuller *t*	0.5466
Unadjusted modified Dickey-Fuller *t*	1.5568*
Unadjusted Dickey-Fuller *t*	1.5638*

Significance level is denoted by *** for 1%, ** for 5%, and * for 10%.

**Table 7:** Estimates of quantile regression Model 1.

**Dep. Var.: *MI***	**Quantiles Model 1**			
	**Q_**0.25**_**	**Q_**0.50**_**	**Q_**0.75**_**	**Q_**0.90**_**
*GHG*	0.794 [1.411]	1.989 [2.138]	1.965** [0.852]	0.819 [0.509]
*GDP*	−2.433 [1.749]	−2.214 [2.652]	−1.989* [1.056]	−0.635 [0.631]
*RQ*	30.255*** [5.752]	6.044 [8.721]	3.352 [3.474]	1.143 [2.074]
*GHG*	−1.255*** [0.436]	−0.141 [0.661]	0.475* [0.263]	0.272* [0.157]
*HC*	−1.259 [1.253]	−0.728 [1.900]	−0.799 [0.757]	−1.242*** [0.452]
*Constant*	2.477 [25.353]	25.204 [38.439]	24.402 [15.311]	8.711 [9.143]

Significance level is denoted by *** for 1%, ** for 5%, and * for 10%.

**Table 8:** Estimates of quantile regression Model 2.

**Dep. Var.: *MC***	**Quantiles Model 2**			
	**Q_**0.25**_**	**Q_**0.50**_**	**Q_**0.75**_**	**Q_**0.90**_**
*GHG*	5.083** [2.448]	4.379 [2.907]	2.906* [1.751]	2.562*** [0.555]
*GDP*	−6.399** [3.036]	−5.008 [3.605]	−2.214 [2.172]	−2.120*** [0.687]
*RQ*	32.001*** [9.984]	26.877** [11.856]	2.247 [7.142]	6.951*** [2.262]
*GHG*	−2.500*** [0.756]	−1.722* [0.898]	0.435 [0.541]	0.306* [0.171]
*HC*	1.929 [2.175]	−1.144 [2.583]	−0.671 [1.556]	−0.461 [0.493]
*Constant*	54.032 [44.008]	41.861 [52.257]	30.183 [31.477]	23.482 [9.968]

Significance level is denoted by *** for 1%, ** for 5%, and * for 10%.

**Table 9:** Dumitrescu-Hurlin panel causality.

***H*_*0*_**	***Wald*_*Stats*_**	** ${\bar{Z}}_{stats}$ **	***p*−*value***
**Model 1**			
*GHG* **–** *MI*	11.6358*	1.75726	0.0789
*MI* **–** *GHG*	5.4221	−0.18020	0.8570
*GDP* **–** *MI*	6.77505	0.24166	0.8090
*MI***–** *GDP*	8.49786	0.77884	0.4361
*RQ* **–** *MI*	3.77864	−0.69263	0.4885
*MI* **–** *RQ*	6.96942	0.30227	0.7624
*GHE* **–** *MI*	10.8832	1.52261	0.1279
*MI* **–** *GHE*	17.8688***	3.70074	0.0002
*HC* **–** *MI*	14.1354**	2.53665	0.0112
*MI* **–** *HC*	78.5401***	22.6184	0.0000
**Model 2**			
*GHG* **–** *MC*	5.12617**	2.45383	0.0141
*MC* **–** *GHG*	1.87940	−0.46859	0.6394
*GDP* **–** *MC*	5.81539***	3.07420	0.0021
*MC* **–** *GDP*	2.86836	0.42158	0.6733
*RQ* **–** *MC*	3.25872	0.77294	0.4396
*MC* **–** *RQ*	2.54951	0.13457	0.8929
*GHE* **–** *MC*	8.11470***	5.14382	3.E-07
*MC* **–** *GHE*	2.34953	−0.04543	0.9638
*HC* **–** *MC*	8.26770***	5.28153	1.E-07
*MC* **–** *HC*	9.60504***	6.48528	9.E-11

Significance level is denoted by *** for 1%, ** for 5%, and * for 10%.

### Slope Heterogeneity and Cross-Sectional Dependence

To understand the slope variation before performing Quantile regressions, slope heterogeneity is applied to examine the variation among the variables in a systematic review. This test is superior to other heterogeneity tests because it allows cross-sectional heterogeneity with large sample periods and small cross-sections that conventional tests lack (59). The test results in Table 2 confirm the presence of heterogeneity in both models. The *t*-statistic values are substantial with a 1% level of significance. This rejects the null hypothesis indicating correlation in both econometric models. These findings lead to the analysis of the cross-sectional dependence of the variables.

Usually, the panel data is subjected to cross-sectional dependence where every unit affects each of them in distinct ways (60). The results of the cross-sectional dependence of the variables are displayed in Table 3. The test statistics show cross-sectional dependence between the research variables, thereby rejecting the null hypothesis. All variables are cross-sectionally significant with positive coefficient values at a 1% significance level except regulatory quality, which has a negative coefficient. The positive significance of variables depicts the relative correlation of variables, which implies that malarial shock tends to spread across countries (emerging).

### Unit Root Test

The unit root determines the stationarity among the variables of the research. Before moving toward cointegration analysis, the unit root is necessary as a pre-test for cointegration or regression analysis. The root is equal to one in the null hypothesis and termed as unit root (61). Due to the presence of cross-sectional dependence, simple (conventional) unit root tests become ineffective and give biased and spurious results; therefore, the Pesaran (49) unit root is applied for cross-sectional panel data analysis. Pesaran's (49) root test gives reliable and stable cross-sectional results, using cross-sectional Augmented Dickey fuller test statistics. The outcomes are displayed in Table 4 of the article. The results describe that all variables are statistically significant at the first difference I(1) with negative values. The higher negative values indicate the stronger existence of the unit root and rejection of the null hypothesis. Gross-domestic product is statistically significant with a negative unit root at first difference with 5% level of significance while all other variables are significant at 1% level of significance.

### Cointegration Tests

Tables 5, 6 show the Pedroni Cointegration and Kao cointegration results for both econometric models. Barbieri (62) stated that these cointegration tests are applied to extend the unit root approach to a multivariate approach. These two cointegration tests are extensions of non-parametric Phillip-Pearson statistics (Pedroni) and parametric Augmented Dickey-Fuller statistics (Kao).

Pedroni extended his procedure of panel cointegration and presented residual-based cointegration tests from 1995 to 2004 with no cointegration null hypothesis under seven different types of tests in which they pool the information concerning the presence of cointegration relationships (52). The statistics include Modified Phillips-Perron, Phillips-Perron, and Augmented Dickey-Fuller. These statistics include complete heterogeneity dynamics and cointegration relationships across panels. The statistic values of Pedroni tests are significant, with 1 and 5% levels of significance signifying the rejection of the null hypothesis (no cointegration). The outcomes from Table 5 confirm the presence of cointegration in both Models (1 and 2) in the long-run association of variables.

Table 6 shows the cointegration outcomes for Panel Kao cointegration tests. These also have the null hypothesis of no cointegration and are called residual-based cointegration tests. These are Augmented Dickey fuller and Dickey-fuller tests, in which the Dickey fuller tests are robust despite the presence of (long-run) parameters (62). The statistics include Modified Dickey-Fuller, Dickey-Fuller, Augmented Dickey-Fuller, Unadjusted modified Dickey-Fuller, and Unadjusted Dickey-Fuller. The Model 1 results of Kao cointegration depict the existence of cointegration with a 1% level of significance, rejecting the null hypothesis of no integration. The second Model also has significant values at 0.10 level except for the Augmented Dickey-fuller statistics. The overall Kao cointegration results for both models portray the presence of cointegration. This implies the existence of a long-run relationship among Greenhouse gas emissions, Gross domestic product, Regulatory quality, Government health expenditures, and Human capital.

### Quantile Regressions

**Figure 1:**
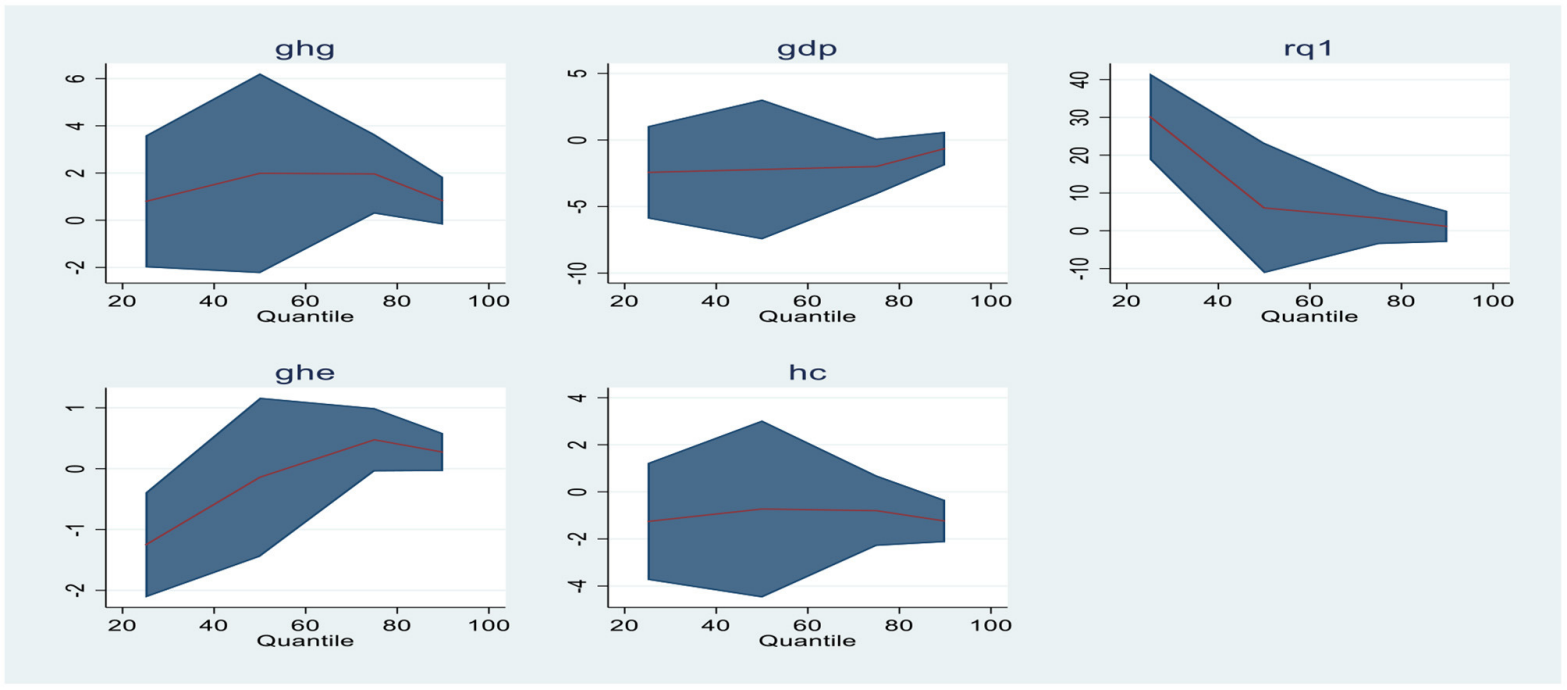
Graphical representation of quantiles for Model 1.

**Figure 2:**
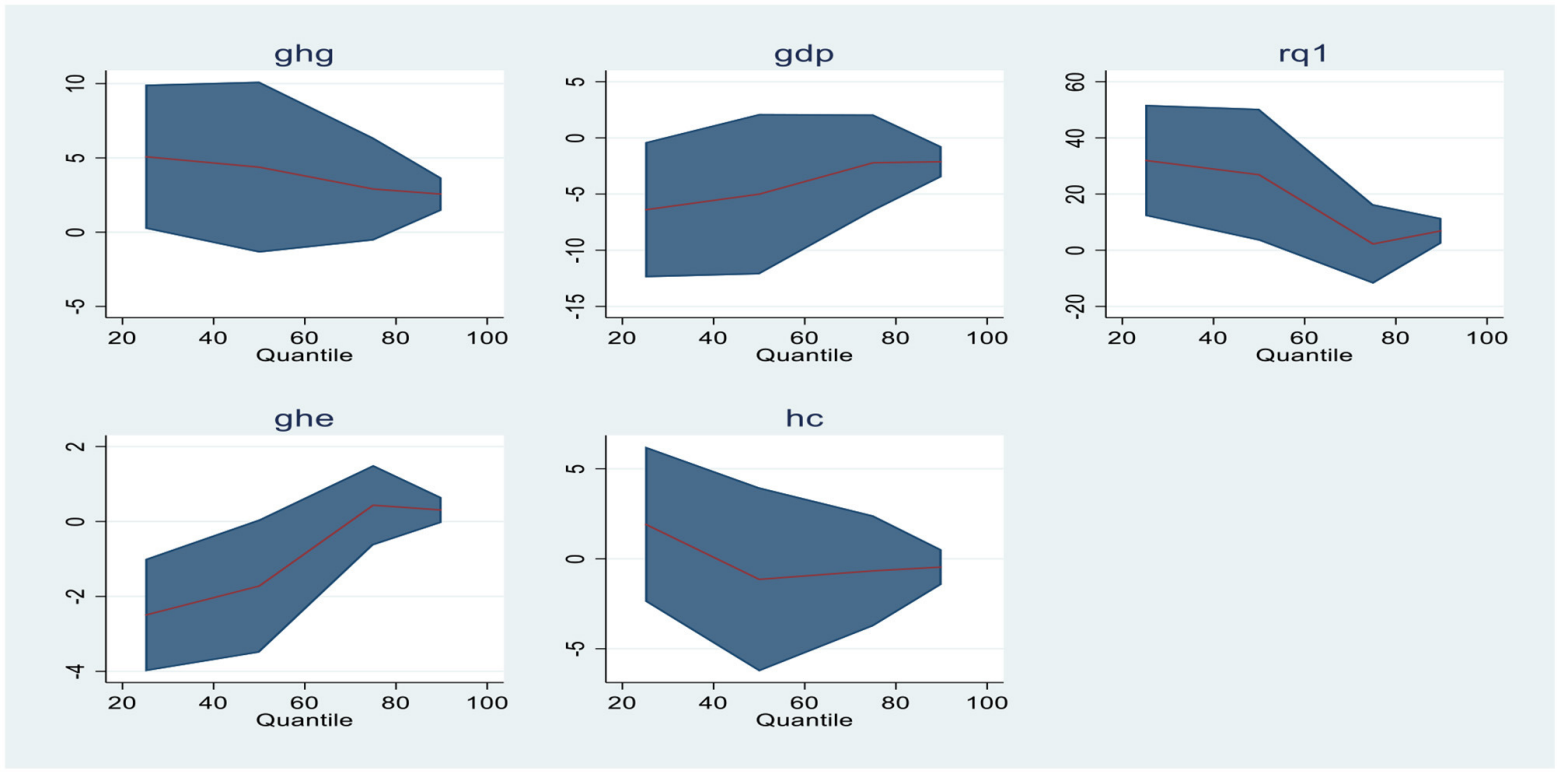
Graphical representation of quantiles for Model 2.

Quantile regressions are extensions of ordinary linear regressions; however, they are usually used when the conditions of linearity are not satisfied and the distribution of residuals is non-normal. These regression analyses were proposed by (53). These regressions are robust toward outliers and give the conditional distributions the finest and most complete picture. The points of distribution are referred to as Quantiles of data. Generally, they are applied either to screen the irregular growth or to examine the determinants of the dependent variable. For instance, in the present study, Quantile regression is applied to explore the role of the explanatory variables for Malaria control or Malaria spread as the dependent variable. Model 1 is used as Malaria incidence (MI), while in Model 2, it is utilized as Malaria cases reported (MC). The outcomes of Model 1 and Model 2 are displayed in Tables 7, 8 below. Figures 1, 2 represent the graphical representation of Quantiles of econometric Models (1 and 2). The graphs of each variable demonstrate the non-linear association between the study's variables.

Table 7 shows the estimates of Quantile regressions for Model 1. The greenhouse gas emissions are significant at the 3rd quantile (Q 0.75) with a 5% significance level. A positive coefficient indicates that increasing GHG emissions has an increasing effect on the dependent variable (MI). Gross domestic product is statistically significant with a 0.10 level of significance at the 3rd quantile. Regulatory quality is significant at 1st quantile with a 0.01 level of significance. Government health expenditures are significant at 1st (Q 0.25), 3rd (Q 0.75), and 4th (Q 0.90) quantiles indicating the significant influence on Malaria incidences. Whereas, Human capital shows significance at the 4th quantile (Q 0.90) with a 1% significance level. The overall results describe that the role of GHG, GDP, RQ, GHE, and HC on Malaria spread is significant in Model 1. Regulatory quality as a proxy of government efficiency and GHG shows a positive impact on the occurrence of Malaria increase. GDP, GHE, and HC are negatively related to Malaria incidences. An increase in these variables will lessen the occurrence of Malaria.

For Model 2, the Quantile regression estimates are presented in Table 8. In this Model, Reported Malaria cases (MC) are the dependent variable. Regulatory quality and government health expenses are statistically significant at 1st (Q 0.25), 2nd (Q 0.50), and 4th (Q 0.90) quantiles. Gross domestic product and greenhouse gas emissions are statistically significant at 1st (Q 0.25) and 4th (0.90) quantiles even though GHG emissions are also significant at 3rd quantile positively. The inclusive estimates demonstrate that greenhouse gas emissions and regulatory quality are positively associated with Malaria cases reported signifying that an increase in these variables has a significant and proportional impact on the malaria cases hype. Even though the gross domestic product and government health expenditure negatively influence the dependent variable, indicating a negative and inverse association. Government health expenses affect the Malaria cases and vice versa.

### Dumitrescu-Hurlin Panel Causality

Dumitrescu-Hurlin panel causality is an innovative Granger causality test for panel data analysis. This causality test allows apprehending the slope heterogeneity of the variables. There are 10 pairs of variables in each econometric model for causality analysis. The panel findings of Dumitrescu-Hurlin causality are displayed in Table 9 of this sub-section of the article. The *p*-values and Wald tests stats for Model 1 indicate that the following pairs GHG–MI, MI–GHE, HC–MI, and MI–HC is significant and reject the null hypothesis that there is no variation or Granger Causality between the slope of these variables. The values are significant at 0.01, 0.05, and 0.10 levels of significance. This implies that GHG granger causes Malaria incidences and Malaria incidences cause government health expenditures. Human capital and Malaria incidences cause granger causality to each other indicative of bi-directional causality. An increase in malaria incidences affects the human capital, and human capital influences the occurrence of Malaria in emerging countries. The causality findings of the second econometric Model (Model 2) demonstrate that the succeeding pairs GHG–MC, GDP–MC, GHE–MC, HC–MC, and MC–HC have shown significant results signifying the Granger causality among the variables. This suggests that GHG causes Malaria cases rise, GDP impacts the cases of Malaria, and government health expenses influence the Malaria cases. At the same time, the other pairs have not rejected the null hypothesis.

The general findings of panel causality reveal a role of greenhouse gas emissions in spreading or causing an increase in Malaria incidence of cases in the emerging economies. Government health expenses play a significant role in managing infectious diseases. In contrast, it also suggests that the rise of an infectious disease leads to an increase in health expenses of the country (40, 44).

### Discussion of Findings

The findings conclude that greenhouse gas emissions and health expenditure are cointegrated in the panel. The heterogeneity and cross-sectional dependence tests demonstrated correlation among the variables. The panel unit root and cointegration analysis confirmed the presence of a long-run relationship. The quantile regressions reported that a significant increase in GHG emissions increases health issues and expenditures on health significantly. Several studies in the literature have stressed that carbon and GHG emissions affect human health and increase health expenditures (30, 39). Effective institutions and policy implementation, together with efficient governments, have a significant and positive influence on improving the health of people (42). The empirical results of the research were consistent with several studies such as (30, 32, 40, 63). These studies examined that environmental degradation and carbon emissions have harmful impacts on individuals' health, leading to an increase in government health expenditure. However, the health expenses were different in different countries but significant in all countries. The authors justified that the negative effect of emissions is due to increased productivity and economic activity and the consumption of non-renewable energy that causes the increasing rate of GHG emissions in the country (64). Moreover, increased consumption of carbon emissions degrades the quality of the environment causing infectious diseases to rise. Increasing utilization of non-renewable energy leads to an increase in greenhouse gas and carbon emissions, which increases the country's health expenditure. One interpretation of the present findings is that poor health infrastructure and a low level of GDP have a substantial influence on rising cases of infectious disease. Poor economy means low per capita income; people will find it difficult to purchase medicines or food for procurement that harms their health, leading to high mortality and morbidity rates and Malarial spread rise, especially in poor economies.

## Conclusions and Policy Implication

To conclude, the increase in greenhouse emissions has increased the Malaria spread across the emerging economies. Notably, this research introduces the role of greenhouse gas emissions, government health expenses, and institutional quality over the spread of Malaria or Malaria control. To examine this, cross-sectional panel cointegration tests and quantile regressions with Dumitrescu-Hurlin Panel Causality tests are applied. The findings of the extant study are quite similar to empirical findings of (30, 32, 40, 43, 44). They emphasized carbon emissions and institutions influence the expenditures of health. However, the present findings indicate that increasing greenhouse emissions have a significant impact on Malaria cases reported and Malaria incidents in the case of emerging economies. The study aims to analyze whether the health expenses, institutional quality, and greenhouse emissions influence Malaria spread in the emerging seven economies, i.e., Brazil, Russia, India, China, Mexico, Indonesia, and Turkey. For this, the study shelters the gap with two econometric models. The first Model utilizes Malaria incidents as a dependent variable, while the second Model (extension and modification of the first Model) utilizes Malaria cases reported as the response variable.

The precise findings of panel causality tests and quantile regressions reveal the role of greenhouse gas emissions in spreading or causing an increase in Malaria incidence or cases in the emerging economies. The government health expense increases with the increase of Malaria. The value of GDP has a negative association with disease spread (cases or incidences). The comprehensive results express that an increase in health expenses is not the only answer for health quality enhancement and disease control. Government and their institutions, NGOs, foreign investment in health projects, awareness programs at the national and international level, and efficient regulatory environmental laws with effective implementation of respective policies can help in reducing the harmful impacts of climate change. The increase in GHG increases health expenses. Additionally, the provision of long-lasting health drugs for malaria control, funds by international organizations to reduce carbon consumption, and educational and awareness programs for infectious disease control combined with preventive measures can help plummet the spread of the diseases and mortality rates in Malaria in emerging economies.

The empirical findings suggest that policymakers must take effective measures to enhance the quality of the environment that mitigate GHG emissions and disease control. First, the governments and international organizations fund those areas or countries where there are high cases of Malaria and make health-sustaining reforms for improving the quality of health and Malaria reduction. Provision of health services to people. Additionally, the expenses must be subsidized or provide free health facilities, especially for Malaria eradication. Second, limit the carbon emissions by transmitting non-renewable energy to renewable energy sources. Immediate and effective action and implementation of transmission programs must be held for environmental and health sustainability. The warming of the earth's temperature due to climate variability creates greenhouse gases that lead to several infectious diseases. The world is potentially facing adverse impacts on human lives. Government, public institutions, and efficient regulatory environmental laws can help reduce the adverse climatic effects.

### Limitation of the Study

Last, of all, the constraint of the study can be expanded for future research. The current study focuses on analyzing the panel quantile regressions to determine the long-run impact of each explanatory variable on human health at four quantiles in the case of emerging economies. Therefore, further research can analyze the influence in other countries especially poor economies with the inclusion of other research variables to determine the impact more deeply. Additionally, the role of urbanization, economic expansion, economic complexity, and private-public investment in the energy sector can be utilized for future research purposes.

## Data Availability Statement

The original contributions presented in the study are included in the article/supplementary material, further inquiries can be directed to the corresponding author/s.

## Author Contributions

All authors listed have made a substantial, direct, and intellectual contribution to the work and approved it for publication.

## Conflict of Interest

The authors declare that the research was conducted in the absence of any commercial or financial relationships that could be construed as a potential conflict of interest.

## Publisher's Note

All claims expressed in this article are solely those of the authors and do not necessarily represent those of their affiliated organizations, or those of the publisher, the editors and the reviewers. Any product that may be evaluated in this article, or claim that may be made by its manufacturer, is not guaranteed or endorsed by the publisher.
